# Non-Coding RNA in the Pathogenesis, Progression and Treatment of Hypertension

**DOI:** 10.3390/ijms19040927

**Published:** 2018-03-21

**Authors:** Christiana Leimena, Hongyu Qiu

**Affiliations:** Department of Basic Sciences, Physiological Division, School of Medicine, Loma Linda University, Loma Linda, CA 92324, USA; cleimena@llu.edu

**Keywords:** non-coding RNA, micro RNA, hypertension

## Abstract

Hypertension is a complex, multifactorial disease that involves the coexistence of multiple risk factors, environmental factors and physiological systems. The complexities extend to the treatment and management of hypertension, which are still the pursuit of many researchers. In the last two decades, various genes have emerged as possible biomarkers and have become the target for investigations of specialized drug design based on its risk factors and the primary cause. Owing to the growing technology of microarrays and next-generation sequencing, the non-protein-coding RNAs (ncRNAs) have increasingly gained attention, and their status of redundancy has flipped to importance in normal cellular processes, as well as in disease progression. The ncRNA molecules make up a significant portion of the human genome, and their role in diseases continues to be uncovered. Specifically, the cellular role of these ncRNAs has played a part in the pathogenesis of hypertension and its progression to heart failure. This review explores the function of the ncRNAs, their types and biology, the current update of their association with hypertension pathology and the potential new therapeutic regime for hypertension.

## 1. Introduction

Hypertension is a major risk factor for the development of cardiovascular disease (CVD). According to a report in 2016 of population-based studies on the global disparities of hypertension, between 2000 and 2010, globally, 31.1% or 1.39 billion people were estimated to suffer from hypertension [1,2]. In 2017, the American College of Cardiology (ACC) and the American Heart Association (AHA) presented new guidelines that further lowered the definition of high blood pressure at 130/80 mmHg rather than 140/90 mmHg, which further highlights the importance of the early detection and intervention of hypertension [3]. There are two major types of systemic hypertension: essential hypertension, which accounts for 95% of all cases, and secondary hypertension [4]. Essential hypertension, also referred to as primary hypertension, is a multifactorial disease where environmental factors and genetic factors coexist. Essential hypertension is characterized by high blood pressure mainly developed at middle or elderly age, while in childhood, essential hypertension is becoming more common due to the obesity epidemic [4]. Secondary hypertension on the other hand has a younger disease onset, with an absence of family history and underlying causes such as endocrine, or renal disorder, or an iatrogenic trigger, or from different medications, including oral contraceptives, steroids, nonsteroid anti-inflammatory drug (NSAIDs) and cyclosporine [5].

The development of hypertension is complex and multifactorial, attributed to both or either of the genetic and/or environmental factors involving at least the renin-angiotensin-aldosterone system, thrombogenesis, impaired platelet function and the sympathetic nervous system [6,7,8,9,10]. The therapeutic drug designs have been based on genes and their encoded proteins that are involved in these signaling pathways. Although pharmacotherapies using various classes of drugs have been shown to have some efficacy in reducing cardiovascular mortality (by 33%), major adverse cardiovascular events (by 29%) and heart failure (by 37%), hypertension remains one of the world’s great public health problems [11]. There is a greater need to further understand the disease mechanism of hypertension and targeted therapeutic treatment [12]. Due to the growing technology of genomics, such as microarrays and next-generation sequencing, the non-protein-coding RNA (ncRNAs) have increasingly gained attention in normal cellular processes, as well as in disease progression. ncRNA is a functional RNA molecule that is transcribed from DNA, but not translated into proteins and has been shown to be involved in regulating gene expression and inhibiting the translation and degradation of messenger RNAs [13]. Two major types of ncRNA, namely microRNAs (miRNAs) and long non-coding RNAs (lncRNAs), have been extensively studied in both hypertensive patients and animal models as outlined in a number of review papers [14,15]. This review will present an update of the most recent progress in both miRNAs and lncRNA focusing on their links to the physiological regulation and therapeutic potential in systemic hypertension. 

## 2. Discovery and Application of Non-Coding RNAs

Since the 1950s, various types of ncRNAs have been uncovered in eukaryotic cells, including transfer RNAs (tRNAs), which make up the greatest number of RNA molecules with 10 tRNAs per ribosomal RNA (rRNA), rRNA, messenger RNA (mRNA), small nucleolar RNA (snoRNA), small nuclear RNA (snRNA), miRNA, the RNA component of the signal recognition particle (7SL RNA), other lncRNAs, circular RNA, heterogeneous nuclear RNA (hnRNA) and X-inactive-specific transcript RNA (Xist RNA) [16,17]. The ncRNAs could be classified based on size: small (around 20 base pairs (bp)), intermediate (less than 200 bp) and long (longer than 200 bp). Small ncRNAs have attracted many investigations such as: piwi-interacting RNAs, small interfering RNAs (siRNAs) and miRNAs [14]. Intermediate ncRNAs include small nuclear RNAs that are involved in splicing during protein synthesis, nucleolar RNAs that modify ribosome RNA, transcription start site (TSS)-associated RNAs and promoter-associated small RNAs [14]. The rest of the ncRNAs that are greater than 200 bp have been grouped as lncRNAs. The research on lncRNAs has gained momentum, and these partake in the epigenetic regulation of transcripts and inactivation of X-chromosomes [14].

Historically, the discovery of tRNA and rRNA began in the 1950s, and the existence of other ncRNA such as snRNAs and 7SL was uncovered from the late 1970s. However, it was in the 1980s that the transcription regulatory function of miRNA began to emerge. It began with the first discovery of micF RNA in *Escherichia coli* [18,19,20]. Following this, in the 1990s, the first regulatory miRNA, lin-4, in eukaryotes was discovered in *Caenorhabditis elegans* (*C. elegans*) [21]. Within the same decade, the lncRNA, Xist, became known as the regulator of the X-chromosome [22]. It was not until in 2000 when the second *C. elegans* miRNA, let-7, was discovered with sequence conservation amongst humans and animals, that the research into miRNAs in *Drosophila* and human cell lines increased exponentially [23,24]. In 2002 was the first report of human miRNAs miR-15a and miR-16-1 that were downregulated or deleted in B cell chronic lymphocytic leukemia cases [25]. Following this finding was the first human oncogenic miRNAs, miR-17-92 cluster and miR-155, which were overexpressed in other cancers and B cell lymphomas, as well as in hematological malignancies, respectively [26,27,28].

The various functions of ncRNAs, in particular, miRNAs and lncRNAs, have unlocked opportunities and developments in clinical trials for RNA interference (RNAi) as the next medical therapy. RNAi medicine is currently via the utilization of siRNAs and miRNA mimics. Strategies of therapeutic design using miRNAs could be in the form of repressing/inhibiting the upregulated miRNA by using antagomirs, which are synthetic antisense 21–23-base pair (bp) oligonucleotides. Alternatively, deficient miRNAs could be replaced or enhanced by the overexpression of miRNAs or utilizing synthetic miRNAs. The antisense technologies have also been trialed to repress lncRNA. The advantages that miRNAs provide over the conventional drug molecules are their potency, action on any gene of interest and accessibility to repression, which some traditional drug molecules could not access due to the encoded protein’s folding conformation and/or, the protein’s lack of enzymatic function [29,30].

There are promising prospects of therapeutic miRNA in the pharmaceutical industry. According to a recent review on therapeutic miRNA and siRNA, there are currently 10 existing miRNA therapeutics in pre-clinical trials [31]. Only one miRNA therapy has proceeded to the phase II clinical trials, miravirsen, utilizing LNA, an antisense oligonucleotide, against miR-122 for hepatitis C infection treatment [32,33]. A therapeutic miRNA, known as MRX34, was developed for cancerous cells, but also other disorders, such as: Alport syndrome, myocardial infarction on remodeling, cardiac fibrosis, abnormal red blood cell production, cardiometabolic disease and chronic heart failure [31]. The MRX34 miRNA therapy is a miRNA mimic, introducing miR-34, which is suppressed in tumor cells. MRX34 therapy is the only one that had entered a phase I clinical trial, but due to the severe adverse immune responses, further progress was halted. The treatment for remodeling in post-myocardial infarction, known as an anti-miR candidate drug named MGN-1374, targets miR-15 and miR-195, has entered into the preclinical stage. For more information on the ongoing or completed clinical trials, the National Institute of Health (NIH), USA, has provided an accessible database (http://www.clinicaltrials.gov).

## 3. Recent Progress of miRNAs in Hypertension

miRNAs (18–24 bp) are master gene regulators controlling the expressions of specific genes by their binding to the 3′ untranslated region (UTR) of a messenger RNA (mRNA), which triggers either repression or degradation of the translation mechanism, and thus gene expression. A single miRNA regulates one to several hundred genes, and a single gene could be regulated by more than one miRNA. miRNAs can be sourced from tissues, urine, serum, plasma and blood cells (which include peripheral blood mononuclear and vascular endothelial cells). The accessibility of miRNA from serum, plasma or urine stems from studies that validated the presence of circulating miRNAs packaged in exosomes, microvesicles or apoptotic bodies [34,35,36,37]. The functions of the circulating miRNAs are mainly to communicate to neighboring or remote target cells and to provide gene expression regulation. An example for this is the communication between endothelial cells and the VSMCs, via the circulating miRNAs in these mentioned extracellular vesicles [34]. The various miRNAs that will be described are also listed in Table 1.

### 3.1. miRNAs in the Regulation of the Renin-Angiotensin Aldosterone System

The renin-angiotensin aldosterone system (RAAS) is a hormonal system that is paramount in the regulation of blood pressure by its influence on cardiac contractility, blood volume and resistance in the vasculature. The RAAS is a collaboration of the physiological workings of various organs and systems: the renal system, the cardiovascular system, the central nervous system and adrenal glands [38]. The RAAS involves a number of molecular players: peptides (angiotensin II), substrate (angiotensinogen (AGT), enzymes (angiotensin converting enzymes 1 and 2 (ACE1 and ACE2, respectively), aldosterone and vasopressin (known as anti-diuretic hormone (ADH)) and receptors (angiotensin II receptor type 1 and type 2 (AT_1_R and AT_2_R encoded by AGTR1 and AGTR2 mRNAs, respectively), bradykinin receptor 2 (B2R) and thromboxane A2 receptor (TBXA2R)). Hypertension develops when this well-balanced system of RAAS is over-activated. A number of microRNAs interact with the major players of RAAS in the hypertensive human cases and animal and in vitro experiments, as shown in Table 1.

It has been shown that many major players of RAAS are regulated by miRNAs. From single-nucleotide polymorphism (SNP) datasets, the *AGTR1* gene, which encodes for angiotensin II receptor type 1, is regulated by miR-155 by its preferential binding to the A allele at position +1166 of the 3′UTR of *AGTR1* [40]. Interestingly, in hypertension cases, there is a higher prevalence for the C allele than the A allele, which reduces the ability of miR-155 to bind to *AGTR1* [40], and individuals who were homozygous for the C allele showed lower miR-155 expression and higher *AGTR1* expression, which resulted in elevated blood pressure [39]. The role of miR-155 in repressing *AGTR1* was tested and confirmed in rat cardiomyocytes, which also reduced cardiac hypertrophy [74]. Furthermore, within a hypertensive cohort, SNPs found in the miRNA binding sites of the RAAS protein genes were associated with elevated blood pressure (in *AVPR1A*) or lower blood pressure (in *BDKRB2* and *TBXA2R*). This finding suggests the role of miRNAs in blood pressure regulation via the genes of RAAS [41]. There has been a collection of reports of SNPs identified in miRNA binding sites, as well the miRNA promoter, and these have modified the binding proficiency of miRNAs to the corresponding target gene and are associated with elevated blood pressure [75,76,77]. The most recent SNP in the 3′UTR gene is found in the Chinese Han population, within the miRNA miR-495 binding site. The mutant C allele has increased the hypertension susceptibility, but further tests are required to determine if this SNP has altered the miRNA binding efficiency [78]. Another report of an association study of hypertensives (156) and normotensives (187) has discovered SNP rs4705342 in the miRNA promoter with a lower frequency of minor C allele among the hypertensive group. However, no further tests have been done to verify the effect of the SNP on miR-143 expression or binding [79]. A list of more of these SNPs that alter miRNA binding sites is provided in Table 2. In addition, microarray data from human, rat and mice showed that miR-483-3p can downregulate AT2R, AGT, ACE1 and ACE2 [42]. Moreover, the cluster of miR-143/145 has been shown to increase under shear stress by activation of the AMPK-p53 pathway, which in turn downregulates the ACE expression [43]. Interestingly, an earlier study has found that expression of miR-143/145 is vital for the maintenance of the VSMC contractile phenotype [80]. A recent study showed that knockout of miR-143/145 in mice resulted in them developing the loss of myogenic tone, and under induction of AngII for increased blood pressure, these knockout mice developed severe vascular inflammation and fibrosis, compared to their wildtype littermates [44].

On the other side, the increase of AngII as a vasoconstrictor that induces the release of aldosterone and vasopressin has also altered miRNA expression. A study on Sprague-Dawley rats showed that 10 days of AngII intravenous infusion induces cardiac hypertrophy and fibrosis and increases miR-132 and miR-212 expression in rat hearts, aortas and kidneys [45]. When applied to the human setting, blocking AngII activity by AGTR1 blocker treatment in hypertensive patients reduces the expression of miR-132 and miR-212 in the internal mammalian artery compared to the control group [45]. The β-blocker drug was also trialed, but the AGTR1 blocker was more potent in its attenuating effect. These studies suggest that miR-132 and miR-212 assist AngII-induced hypertension. Within the in vitro setting, AngII treatment in human adrenocortical cell lines increases miR-21 expression. This releases aldosterone secretion, but not cortisol. This result suggests the possibility that miR-21 could influence the abnormal aldosterone secretion in the hypertension setting and contribute to primary aldosteronism [46].

A number of studies has reported changes in the expressions of miRNAs and its possible use as a biomarker in connection with sodium homeostasis, blood pressure and renin expression. In a small cohort study (*n* = 10) that evaluated, using a microarray, the correlation of salt intake and blood pressure, 45 differentially-expressed miRNAs were found with miR-4516 displaying the highest expression change across salt intake variation [47]. Interestingly, the exosomes from the urine samples showed a reduction of miR-4516 expression in the inverse salt-sensitive (mean arterial pressure (MAP) decreases ≥7 mmHg with high salt intake) vs. the salt-resistant subjects (control; <7 mmHg MAP change with high salt intake) and, conversely, an increase in the expression in the salt-sensitive (≥7 mmHg increase in MAP) vs. the salt-resistant group [47]. Similarly, a recent report showed other miRNAs, miR-361-5p and miR-362-5p, being associated with salt sensitivity. Both of these miRNAs were downregulated in the salt-sensitive hypertensive group, compared to the salt-resistant essential hypertensive group [48]. Within another cohort of white hypertensive European subjects, microarray profiling on the medulla and cortex of kidney tissues along with qPCR validation showed: downregulation of miR-638 and let-7c, in the medulla, and in the renal cortex, downregulation of miR-181a, miR-638 and miR-663 and upregulation of miR-21, miR-126, miR-196 and miR-451 [49].

The above findings of the downregulation of miR-181a and miR-663 in the hypertensive human cohort can be correlated further to some in vitro and animal studies. In the human embryonic kidney cell cline (HEK293), miR-181a and miR-663 regulate the endogenous renin expression by targeting the 3′UTR of the human renin (REN) mRNA, and specifically also apoptosis-inducing factor mitochondrion-associated 1 (AIFM1) and apolipoprotein (APO E), respectively [49]. In the BHP/2J mouse circadian hypertension model [50], they found downregulation of miR-181a and increased renin expression in the active period [50].

### 3.2. miRNAs in Endothelial Dysfunction

Vascular endothelial dysfunction is highly associated with hypertension. Endothelial cells function to release vasodilators into the blood stream to reduce vascular resistance. As these cells face continuous hemodynamic forces with shear stress and stretch, they play an important role in development, regulation and remodeling of the vasculature. Increased blood pressure can alter the phenotype and function of the endothelial cells [82]. With endothelial dysfunction, there is a reduced vasodilatation, activation and release of inflammatory factors, an increase of reactive oxygen species (ROS), a reduction of nitric oxide, which then develops into increased vascular tone, increased vascular stiffness and pulse pressure, and sustained elevated blood pressure [83]. The maintenance of the endothelial cell phenotype involves a number of molecular players such as protein kinases, integrins, endothelial nitric oxidase synthase (eNOS) and NO, vascular endothelial growth factor (VEGF) and miRNAs [84,85]. Evidence has emerged that miRNAs are involved in angiogenesis, the proliferation and function of endothelial cells and their dysfunction.

Several human studies have shown the association of miRNAs and hypertension in the aspect of endothelial dysfunction. In experimental models, change in expression of an l-arginine transporter gene, *SLC7A1*, alters NO production and induces endothelial dysfunction. An SNP of a novel C/T polymorphism in the 3′UTR of *SLC7A1* is found in 278 essential hypertensive subjects (T allele frequency: 13.3%), compared with the 498 normotensive subjects (T allele frequency: 7.6%) [51]. The impact of the T allele is such that it disrupts the binding of transcription factor SP1, extends the 3′UTR length to increase the binding site of miR-122, which in turn reduces *SLC7A1* expression. A tumor-suppressive miRNA, miR-505, was found to be increased in hypertensive patients from three independent cohorts [52]. The increase of miR-505 represses fibroblast growth factor 18 (FGF18), a proangiogenic factor in the endothelial cells that promotes endothelial migration and thus disables the migration and tube formation of endothelial cells [53]. Another study shows that vascular endothelial cells release circulating miRNAs to combat pathogenic virus. From plasma collection of a hypertensive and normotensive Chinese population, three significant miRNAs were isolated: human cytomegalovirus (HCMV) miRNA (hcmv-miR-UL112), miR-296-5p and let-7e. Based on in vitro transfection of HEK293 cells with the reporter gene constructs, the interferon regulatory factor 1 has been reported to be the target of hcmv-miR-UL112 [54]. Interestingly, hypertensive patients showed increased HCMV seropositivity and quantitative titers (52.7% vs. 30%, *p* = 0.0005; 1870 vs. 54 copies per 1 mL plasma, *p* < 0.0001), which suggest that cytomegalovirus causes the release of hcmv-miR-UL112 from vascular endothelial cells. The group suggested a possible new link between HCMV infection and essential hypertension [54].

There is some evidence of the involvement of miRNA and eNOS production in hypertension and in endothelial dysfunction. eNOS is responsible for the production of NO within the endothelium. Inhibition of eNOS decreases NO availability and increases oxidative stress, endothelium dysfunction and hypertension. A study reported that inflammatory release of tumor necrosis factor-α (TNF-α) induces the transcription of miR-155, which targets the 3′UTR of eNOS to inhibit its expression. Inhibition of miR-155 reverses the effect of eNOS downregulation [55]. Furthermore, the miR-221/222 cluster, known as the sensitive regulator in the endothelium, regulates the NO release by its binding to the 3′UTR of eNOS mRNA in endothelial cells, as well as other genes, such as activator STAT5a, transcription factors Ets1, Es2 and cyclin-dependent kinase cell cycle regulators p21^Cip1^ and p27^Kip1^ [56,57,58]. Recently, it was found that the presence of pro-inflammatory cytokines upregulates miR-146a and miR-146b. Their upregulation not only inhibits endothelial activation and represses NF-κB and MAP kinase pathways, but also targets HuR, which is an RNA binding protein that suppresses eNOS [59].

Interestingly, Dicer, the RNaseIII enzyme, not only plays a role in processing the premiRNA sequence into a shorter double-stranded miRNA, but also contributes to angiogenesis. The absence of Dicer in both the in vitro and in vivo system causes dysregulation in angiogenesis. There is a number of miRNAs that are also involved in angiogenesis. One of them is the endothelial-specific miR-126. The role of miR-126 includes: influencing the integrity and regulation of the growth of the vasculature by controlling the endothelial response to VEGF, inhibiting the negative regulators of the VEGF pathway and controlling the level of the vascular cell adhesion molecule (VCAM-1) for inflammatory adhesion [60,61,62].

### 3.3. miRNAs Involved in VSMCs and Other Cells in Hypertension

Understanding the role of vascular smooth muscle cells (VSMC) and the factors that influence their function is important for clarity on the pathogenesis and treatment of hypertension. Within the blood vessels, there is a complex interplay of neurotransmitters, circulating hormones and endothelium-derived factors for vasodilation and vasoconstriction. VSMC influences the vascular tone and regulates blood pressure, vascular resistance and tissue perfusion. Various antihypertensive agents have been designed to target the VSMCs (such as the ACE inhibitor, calcium channel blockers) [86]. miRNAs have been found to be involved in the function of VSMC, the development of arterial stiffness and the progression to hypertension [87]. The miRNA miR-21, which has been known to regulate arterial fibrosis, was reported to have a correlation with improvement in arterial stiffness [88,89]. A study with 95 essential hypertensive patients that underwent antihypertensive treatment showed a negative correlation between miR-21 and the pulse wave velocity readings [63]. A study involving 89 individuals, of which 60 had essential hypertension and 29 were normotensive [64], showed that lower miR-143, miR-145 and miR-133, but higher miR-21 and miR-1 were found in peripheral blood mononuclear cells from the hypertensive group, compared to the normotensives. Negative correlation of the diastolic blood pressure (DBP) was found with miR-143, miR-145 and miR-21, but there was a positive correlation with miR-133. Interestingly, the miR-145 expression level was also overexpressed in 22 human atherosclerotic plaques (15 hypertensive and seven control) [90]. Furthermore, miR-145 was reported to have dual role in its binding to TGFβ receptor II (TGFBR2) [65]. The modulation of TGFβ receptor 2 signaling affects the downstream expression of the matrix genes in VSMC [65]. The same group that investigated the hypertensive cohort also compared the expression of miR-9 and miR-126 in peripheral blood mononuclear cells between the hypertensive and normotensive group [66]. There was a significant lower expression found in both miR-9 and miR-126 in the hypertensive group, and their expression level showed positive correlation with the 24-h mean pulse pressure [66]. The expression level of miR-9 showed a positive correlation with the left ventricular mass index. Another study reported that both miR-126 and miR-223 are involved in regulating vascular inflammation by repressing vascular cell adhesion molecule-1 (VCAM-1) and intercellular adhesion molecule-1 (ICAM-1), respectively [67,68]. The VSMC proliferation has also been found to be influenced by the level of miR-34b. Through qPCR, in silico analysis and the luciferase assay, miR-34b was found to target cyclin-dependent kinase 6 (CDK6), which controls cell cycle progression and proliferation [69].

Recently, a number of miRNAs has been detected to be differentially expressed in essential hypertensive individuals, compared to the healthy individuals. Increased expression of circulating miR-29a/b/c and miR-510 by qPCR was found in hypertensive individuals, compared to the normotensive individuals [70,71]. Another group has reported two sets of data of the upregulation of miRNAs: let-7 and miR-92a, in correlation with the increase in carotid intima-media thickness (CMIT), compared to the normal CMIT [72,73]. This shows that the miRNA levels could reveal the development of subclinical atherosclerosis with the thickening of the CMIT. Thus, here is another piece of evidence for the possibilities of miRNAs to be used as biomarker and in this case for the detection of end-organ damage in hypertension.

**Figure 1:**
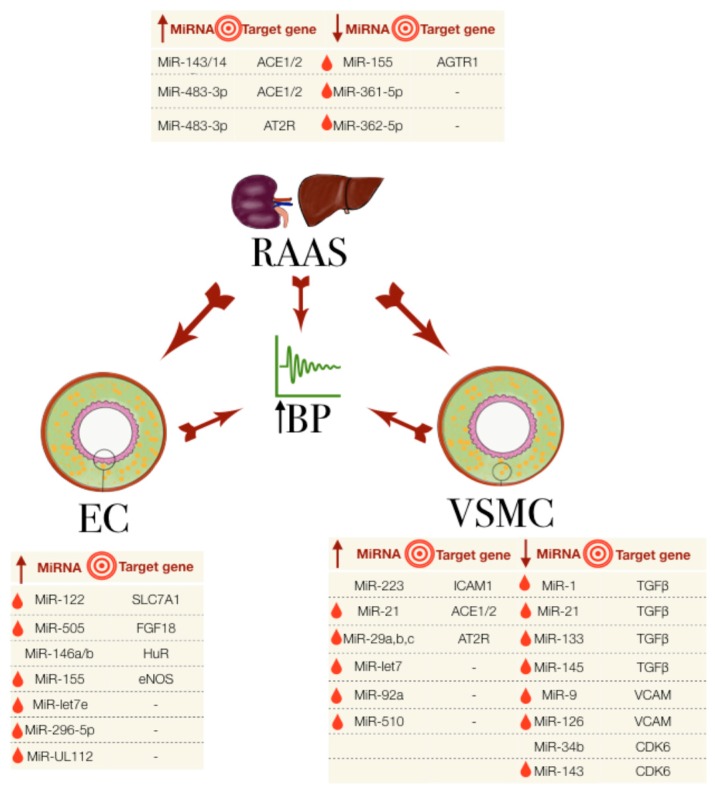
The miRNAs involved in essential hypertension, their association with the RAAS, EC and VSMC and their known target genes. The big central arrows indicate a system/cell type’s influence on another and on the increase in blood pressure (BP). Small arrows in the tables indicate the upregulated (up arrow) or downregulated (down arrow) miRNA expression. Red droplets represent the biomarker potential from detectability in blood samples. EC: endothelial cells; RAAS: renin-angiotensin aldosterone system; VSMC: vascular smooth muscle cells.

A summary of the miRNAs associated with essential hypertension is further tabulated based on species (see Table 3), and the essential miRNAs with their targets are presented in Figure 1.

## 4. Recent Progress of lncRNAs in Hypertension

lncRNAs are typically greater than 200 bp in length. Though they are transcribed, 3′polyadenylated, 5′ capped and spliced, lncRNAs do not translate into proteins. There are four different types of lncRNAs based on their relative genomic location to the coding region, including: intergenic lncRNAs (or lincRNAs), intronic lncRNAs, sense lncRNAs and antisense lncRNAs [14]. Unlike miRNA, lncRNAs have numerous functions in regulating gene expression with transcription and translation (upregulating and downregulating), in splicing, imprinting and cell cycle development. lncRNAs can silence multiple genes through their interaction with chromatin, or even recruit promoters to a target gene and induce transcription. lncRNAs could also be detected in urine and blood, providing a promising future as biomarkers for disease [92].

Although the molecular mechanisms of lncRNAs are not fully understood, some studies showed that they play a role in normal physiology, as well as the development of hypertension and cardiovascular diseases. Recent studies showed that there were 68 lncRNA upregulated and 167 lncRNAs downregulated in spontaneously hypertensive rats (SHR) compared to their normotensive control (Wistar-Kyoto (WKY) rats) [93]. One particular lncRNA, XR007793, was validated to be upregulated in vitro in VSMC of hypertension. Reciprocally, knocking down of XR007793 attenuated VSMC proliferation and migration. Absence of XR007793 also inhibited interferon regulatory factor 7 (irf7), signal transducers and activators of transcription 2 (stat2) and LIM only domain 2 (limo2) [93]. Furthermore, the different expressions of 749 lncRNAs were identified between Dahl salt-sensitive vs. spontaneously hypertensive rats [94]. From these, four candidate target lncRNA-mRNA-associated genes were selected: Ankyrin repeat and SPCS box containing 3 (Asb3), cation transport regulator homolog 2 (Chac2), peroxisomal membrane 11B (Pex11b) and Sp5 transcription factor (Sp5) [94]. The lncRNAs that are specific for these genes were upregulated, while the protein of these candidate genes was downregulated. Recently, lncRNA AK098656 was detected to be upregulated in the plasma of hypertensive patients [95]. This lncRNA mediates the VSMC synthetic phenotype, which is a common characteristic in hypertension pathophysiology. A human genome-wide association study (GWAS) presented a strong association between systolic mean arterial blood pressure and lncRNA H19 locus [96]. The H19 lncRNA is expressed only during embryonic development, but it is upregulated in cardiovascular conditions [96]. In addition, a group genotyped a hypertensive vs. a normotensive cohort for SNPS that were found in a long non-coding RNA, CDKN2B-AS1 [81]. CDKN2B-AS1 is noted to contribute in some ways to regulating the cell cycle and senescence. The genotyping performed in this population showed a significant difference in the genotype frequency of the SNPs between the hypertensive and normotensive groups and strong association between rs10757274 and rs2383207 (AA) and SBP [81].

MALAT1 [97] has been reported to control vessel growth and endothelial cell function. A vascular cell-rich lncRNA, SENCR, has been shown to play a part in the smooth muscle cell phenotype [98]. Furthermore, growth arrest-specific 5 (GAS5) was found to regulate artery remodeling in caudal, renal, thoracic and carotid arteries [99]. GAS5 was also found to be downregulated, which affected endothelial proliferation and activation in hypertensive condition in the rats [99]. Interestingly, a study reported on the effects of goji berries (*Lycium barbarum* L.) on lncRNA in rats with a high salt diet. The consumption of the berries ameliorated the hypertensive condition on the borderline hypertensive rats and reduced the sONE lncRNA, which then reciprocally improved the eNOS expression [100]. The lncRNAs that have been found to be associated with essential hypertension are summarized in Table 4.

## 5. Detection of Non-Coding RNAs

miRNAs and lncRNAs have the potential to be biomarkers and targets for therapy design. Drug design on miRNA and lncRNA replacement, modulation and enhancement is a promising outlook. In the last decade, studies have reported that miRNAs and lncRNAs are packaged in microvesicles, exosomes, apoptotic bodies, high-density lipoproteins (HDL) or Ago2 as RNA-binding proteins and carried in the bloodstream [34,35,36,37,92,101,102]. This makes the miRNAs protected from degradation by RNase activity in the blood. The functions of these circulating ncRNAs are mainly to communicate to neighboring or remote target cells and to provide gene expression regulation. An example for the existence of the transfer of miRNAs in extracellular microvesicles is the communication between endothelial cells and the VSMCs of miRNAs such as miR-143 and miR-145 [34]. The facts that miRNAs and lncRNAs can be detected in circulation (serum and plasma) and urine and that they are stable in blood during transportation and storage enable sample collection to be simpler, faster and non-invasive with no requirement of tissue collection. Despite this promising advantage, more research is required to detect the origin (cell/tissue type) of these circulating miRNAs.

## 6. Non-Coding RNAs in the Treatment of Hypertension

As mis-expression or mutation of miRNAs has been implicated in various diseases, including hypertension, treatments utilizing ncRNAs are still in their infancy. However, there is a promising outlook with the progress in understanding their molecular mechanism. In an animal experimental model, miR-22 antagomir (LNA oligonucleotide) was administered intraperitoneally in SHR and WKY rats [91]. This administration reduced the SBP and DBP by about 18 mmHg in the SHR. Furthermore, in human microRNAome screening, miR-25 was significantly upregulated, and its upregulation delays calcium uptake kinetics specifically for SERCA 2a, in both human and mice failing cardiomyocytes [103]. When miR-25 was overexpressed by in vivo administration using adeno-associated virus 9 (AAV9), there was a confirmation of the loss of contractile function. Interestingly, using antagomirs against miR-25 in a mouse model, cardiac function improved with increased survival compared to the animals that received a control antagomir oligonucleotide [103]. According to a previously-mentioned review on miRNA therapeutics, there are two therapeutic miRNAs correlated with vasculature disorders that are currently in the pipeline of the development phase. The miRagen Therapeutics company has in the pipeline MGN-2677 for the treatment of vascular disease, which involves miR-143/145 [31]. For peripheral arterial disease treatment, mirage Therapeutics utilizes the function of miR-92 for the MGN-6114 therapy design [31].

lncRNAs have not fallen behind with respect to the interest in their use for therapeutic purposes. To overexpress lncRNA, adeno-associated viral vectors have been selected due to their low pathogenicity [104]. The inhibition or downregulation of cytosolic lncRNA has been explored using siRNA or aptamers with antisense oligonucleotide (ASO)-mediated knockdown [105,106,107]. Nuclear lncRNAs, on the other hand, could be downregulated using the GapmeRs system, by heteroduplex formation with the target lncRNAs for cleavage by RNase H [108,109]. 

Although the accessibility of these ncRNAs in circulation has promising biomarker potential, their efficiency and safe delivery for therapy still remain as challenges. The drug design utilizing these small RNA molecules has so far met the challenges of low serum stability, non-specific targeting, innate immune responses and poor pharmacological properties. Developments have emerged in the design of various delivery systems for greater bioavailability: biodegradable polymers, PEGylated liposomes and lipidoids [31]. Furthermore, the vesicles (50–500 nm) have been designed for protection from the kidney filtering system for more efficient intracellular delivery [110].

Despite the promising outlook for therapy utilizing the biology of miRNA, there are some limitations that need to be overcome and considered. As previously mentioned, a single miRNA can target several other genes, and the suppression of one gene may/may not be by more than one miRNA. A treatment using one miRNA may affect other genes that need not be dysregulated in their expression. Development of techniques for detecting microvesicles membrane markers to determine where the circulating miRNAs came from will be useful to ascertain the cell/tissue source of the miRNA. Furthermore, there are racial differences in the miRNA profiling in the disease and non-disease state. A recent study profiled the miRNA expression between the hypertensive and normotensive African American and white American cohorts [111]. There were significant mRNA/miRNA pair expression differences between the AA and white female hypertensives.

## 7. Conclusions

Owing to whole genome sequencing and RNA sequencing technologies, greater information of the non-coding sequences in the genome has been uncovered. Various pharmacotreatment strategies have been employed for hypertension, but due to the complexity of the disease, there still exists room for greater understanding of the disease mechanism and better therapeutic drug treatment. There are more studies that have been performed to elucidate the role of miRNAs in normal vasculature, the development of hypertension and cardiovascular disease. The current findings of miRNA have shown that there is a promising way to modulate the miRNA expression and its repressing action. The understanding of lncRNA is still in its infancy and requires greater work to understand its molecular mechanism. However, both miRNAs and lncRNAs could be detected in urine, blood and plasma, which allows them to be used as biomarkers for disease diagnosis. More research is needed to overcome the listed limitations for the miRNA mechanism for usage in future therapy. 

## Figures and Tables

**Table 1:** miRNAs associated with hypertension.

miRNA	miRNA Expression	Species	Conditions/Treatment	Sample Size	Source	Ref.
**RAAS**						
miR-155	Down	Human	young HT, reporter silencing assay	*n* = 19–25	Blood; HEK293T cells	[39,40]
miR-526, miR-578, miR-34a, miR-34c-5p, miR-449b, miR-571, miR-765	Up	Human	SNP genotyping on miRNA binding sites in genes of RAAS that influence blood pressure	*n* = 1246	Blood, HUH7/HELA cells	[41]
miR-483-3p	Up	Human, rats, mice	MiRNA array, reporter luciferase assay	-	HASMC, RASMC, HL-1 cells	[42]
miR-143/145	Up	Mice	Shear stress on EC of Ampkα2^−/−^ mice	-	EC	[43]
	-	Mice	MiR-143/145 KO mice: AngII-infusion for vascular injury		Mesenteric arteries	[44]
miR-132, miR-212	Up	Rats	AngII-infused and Endothelin	*n* = 3–5	Heart, aorta, kidney	[45]
	down	Human	AGTR1 blocker treatmt	*n* = 16	Artery	[45]
miR-21	Up	human	AngII-induced cells		Cell line	[46]
miR-4516		Human	HT iSS/SS/SR	*n* = 3–4	Exosomes in urine	[47]
miR-361-5p, miR-362-5p	Down	Human	SSH vs. SRH	*n* = 6	Whole blood	[48]
miR-638,181a,663, let-7c	Down	Human	qPCR on HT/NT	*n* = 16–22	Renal medulla	[49]
miR-21,126, 196a,451	Up					
miR-181a	Down	Mouse	Effect of RAAS on hypertension in BPH/2J mouse circadian HT	*n* = 7–13	Kidneys	[50]
**Endothelial cells**						
miR-122	Up	Human	HT	*n* = 278–498	Blood	[51]
miR-505	Up	Human	HT	*n* = 11–19	Plasma, HUVEC	[52,53]
miR-UL112, 296-5p, let-7e	Up		Microarray, qPCR	*n* = 67–127	Plasma	[54]
miR-155	Up	Human	-	*n* = 6	HUVEC	[55]
miR-221/222			Dicer silencing by siRNA on HUVEC, hy.926 cells			[56,57,58]
miR-146a/b	Up	Human, mice	miR 146a^−/−^ mice exposed to inflammatory cytokines	-	HUVEC	[59]
miR-126	-	Mouse / Zebrafish	miR-126^−/−^ mice	-	Stem cells, zebrafish	[60,61,62]
**VSMC and other cells**						
miR-21	Up	Human	HT patients and post antihypertensive treatment	*n* = 95	Peripheral blood mononuclear cells	[63]
miR-143,	Down	Human	Expression analysis of miRNAs involved in VSMC plasticity	*n* = 29–60	Blood cells	[64,65]
miR-145,miR-133	Down	Human				
miR-21, miR-1	Up	Human				
miR-9,126	Down	Human	HT	*n* = 29–60	Blood cells	[66]
miR-126	Up	Human	HUVEC	*n* = 6	HUVEC	[67]
miR-223	Up	Human	High density lipoprotein		HCAEC	[68]
miR-34b	Down	Rats	SHR vs. Wky	*n* = 36	VSMC	[69]
miR-29a/b/c	Up	Human	Untreated essential hypertension vs. healthy individuals	*n* = 30–54	Plasma	[70]
miR-510	Up	Human	HT vs. NT	*n* = 208–220	Blood	[71]
let-7	Up	Human	Expression of let-7 in HT vs. NT with normal/increased CMIT	*n* = 60	Plasma	[72]
miR-92a	Up	Human	Expression of miR-92a in HT vs. NT with normal/increased CMIT	*n* = 60	Plasma	[73]

Abbreviations. HT: hypertensive; NT: normotensive; ISS: inverse salt sensitive; SS: salt sensitive; SR: salt-resistant; SNP: single nucleotide polymorphism; RAAS: renin-angiotensin aldosterone system; VSMC: vascular smooth muscle cells; EC: endothelial cells; KO: knockout; SSH: salt sensitive hypertension; SSR: salt sensitive resistance; HDL: high density lipoprotein; HCAEC: human coronary artery endothelial cells; HEK293T: human embryonic kidney 293T; HUVEC: human umbilical vein endothelial cells; PWV: pulse wave velocity; Wky: Wistar-Kyoto rats; SHR: spontaneous hypertensive rats; n/iCMIT: normal/increased carotid intima-media thickness; BPH/2J: hypertensive blood pressure mice; BPN/3J: normotensive blood pressure mice.

**Table 2:** SNPs associated with hypertension.

SNPs	ncRNA	Gene	SNP site	Ref.
rs3749585	miR-495	CORIN	miR-495 site	[78]
rs10757274, rs2383207, rs10757278, rs1333049	CDKN2B-AS1 (lncRNA)	-	9p21.3	[81]
rs4705342	-	-	miR-143 promoter	[79]
rs17228616	-	ACHE	miR-608	[77]
rs5068	-	NPPA	miR-425 site	[76]
rs938671	-	ATP6V0A1	miR-637 site	[75]
rs5186 (A1166C)	miR-155	AGTR1	miR-155 site	[39,40]
rs11174811	miR-526, miR-578	AVPR1A	miR-536, miR-578 sites	[41]
rs5225, rs2069591	miR-34a, miR-34c-5p, miR-449b	BDKRN2	miR-34a, miR-34c-5p, miR-449b sites	
rs13306046	miR-571, miR-765	TBXA2R	miR-571, miR-765 sites	
ss52051869	miR-122	SLC7A1	miR-122 site	[51]

**Table 3:** miRNAs associated with hypertension based on species.

Species	miRNA	SNPs/Target Gene	Subject/Model	Ref.
Human	miR-155	AGTR1: rs5186 (A1166C)	qPCR on blood mononuclear cells from 64 HT (AA: 25; AC: 20; CC: 19); HUVEC cells	[39,55]
		-	Reporter silencing assay on HEK293T	[40]
Human	miR-638, -181a, -663, let-7c	-	Microarray. Validated by qPCR. Functional studies with HEK293 cells. qPCR HT vs. NT	[49,64,65]
Human	miR-21, -126, -196a, -451	-		
Human	miR-145,133	TGF-β	qPCR HT vs. NT	[64,65]
Human	miR-122	SLC7A1: ss52051869	Genotyping, sequencing, in vitro on HT	[51]
Human	miR-505	FGF18	qPCR HT vs. NT from plasma, luciferase reporter assay	[52,53]
Human	miR-UL112,296-5p,let-7e		Microarray and validated by qPCR on HT vs. NT	[54]
Human	let-7	-	qPCR on let-7 in HT vs. NT with normal/increased CMIT	[72]
Human	miR-155	eNOS	qPCR and in vitro assay on HUVEC	[55]
Human	miR-143	-	qPCR HT vs. NT	[64]
Human	miR-9,126	VCAM-1, ICAM-1	qPCR HT vs. NT	[66]
Human	miR-126	VCAM	Microarray, northern blot and fucntional assay on HUVEC	[67]
Human	miR-223	ICAM-1	Whole genome and miRNA microarray on HDL treated HCAEC, qPCR, luciferase reporter assay	[68]
Human	miR-361-5p, miR-362-5p	-	qPCR on SSH vs. SRH	[48]
Human	miR-21	-	1.HT patients and post antihypertensive treatment. 2 AngII-induced H295R cells	[63]
			AngII-induced H295R cells and luciferase reporter assay	[46]
Human	miR-29a/b/c	-	untreated essential hypertension vs. healthy individuals	[70]
Human	miR-510	-	qPCR on HT vs. NT	[71]
Human	miR-92a	-	qPCR on miR-92a in HT vs. NT with normal/increased CMIT	[73]
Human	miR-4516		qPCR from exosomes of urine of HT ISS/SS/SR	[47]
Human	miR-221/222	eNOS, STAT5a, Ets1, Ets2, p21Cip1, p27Kip1	Mcroarray, Northern blotting on Dicer silenced HUVEC and and EA.hy.926 cells	[56,58]
Human, rats, mice	miR-132, 212	-	Microarray. Validated by qPCR. Humans treated: AngII blocker, β-blocker; rats treated with endothelin, mice treated with AngII	[45]
Human, rats, mice	miR-483-3p	AT2R, AGT, ACE1, ACE2	miRNA array, luciferase reporter assay on HASMC, RASMC, HL-1 cells	[42]
Human, mice	miR-146a/b	HuR	qPCR and intro assay on HUVEC and mice tissues induced by inflammatory cytokines	[59]
Rats	miR-34b	Cdk6	qPCR on SHR vs. Wky	[69]
Rats	miR-22	Chga	Luciferase reporter assay, miR-22 antagomir	[91]
Mice	miR-143/145	ACE	Shear stress on EC of Ampkα2^−/−^ mice, qPCR. MiRagen Therapeutics: MGN-2677	[31,43]
Mouse	miR-181a	-	qPCR on BHP/2J mouse circadian HT	[50]
Mouse/Zebrafish	miR-126	VCAM1, SPRED-1, PIK3 regulatory subunit-2	miR-126^−/−^ mice, mouse ES cells, antisense to miR-126	[60,61]

**Table 4:** lncRNA associated with essential hypertension.

Species	lncRNA	Cohort/Model	Function	Detection/Evaluation	Outcome	Ref.
Human	CDKN2B-AS1	HT vs. NT (Turkish)	Interacts with PRC1 & PRC2 to repress CDKN2A/B locus. Regulate VSMC stiffness	qPCR to test if published 9p21.3 SNPs are associated with BP	Significant difference in genotype freq of the 4 SNPs betw HT and NT. Association betw rs10757274 & rs2383207 (AA) and SBP.	[81]
Human	H19	87,736 indiv. + 68,368 indiv. from European ancestry	Regulator of mammlain development, inhibits cell proliferation. Methylation of H19 associated with preeclampsia and imprinting syndrome and growth retardation.	Discovery meta analysis, genome-wide SNP genotype	11 Loci with 31 genes uncovered with H19 as a lncRNA.	[96]
Human/Rat	GAS5	Transfecton of HUVEC, human VSMC, GAS5 viral knockdown in SHR vs. Wky	Regulate remodelling of arteries (caudal, carotid, renal and thoracic); regulate transcription of androgen, progesterone, mineralcorticoid receptors; involved in cellular growth arrest and apoptosis	BP measurement, tissue staining for arterial remodeling evaluation, qPCR for GAS5 expression	GAS5 expression down regulated in HT. knockdown increased SBP and DBP and mean arterial BP (in SHR) retinal neovascularization and capillary leakage, endothelial activation and proliferation	[99]
Human/Rat	AK098656	HT vs. NT (China); AK098656 transgenic rat model	Induce VSMC synthetic phenotye. Bind to myosin heavy chain-11, fibronectin-1, 26S proteasome non-ATPase regulatory subunit 11, actin, actin-binding protein	LncRNA microarray, whole-genome microarray	Upregulated in plasma of HT group vs. NT, increase VSMC proliferation & migration, upregulate extracellular matrix but downregulate contractile proteins.	[95]
Human/Mouse	MALAT1	HUVEC and MALAT1 KO model	Control cellular proliferation through histone modification	RNASeq, Microarray, qPCR	Vessel growth, endothelial cell function	[97]
Rats	XR007793	Wky/SHR and VSMC subjected to hypertensive level cyclic strain	No known predicted target	Microarray and qPCR	Kncockdown of XR007793 repress VSMC proliferation & migration. Reduced transcript expression of stat2, lmo2 and irf7.	[93]
Rats	749 lncRNAs	Dahl SS/SR and SHR	-	RNASeq, mRNA trasncrptome analysis	Asb3, Chac2, Pex11b, Sp5	[94]
Rats	sONE	Borderline hypertensive rats (BHR) fed high, medium and low salt diets	From transcription unit (NOS3AS) on opposing strand of human eNOS. Inhibiton of sONE increases eNOS and vice versa when sONE is overexpressed.	qPCR	Lycium Barbarum L. ameliorated hypertension, reduced sONE expression and improved eNOS expression compared to high salt diet rats.	[100]

Abbreviations. HT: hypertensive; NT: normotensive; SS: salt sensitive; SR: sat-resistant; SNP: single nucleotide polymorphism; VSMC: vascular smooth muscle cells; KO: knockout; SS: salt sensitive; SR: salt resistant; HUVEC: human umbilical vein endothelial cells; PWV: pulse wave velocity; Wky: Wistar-Kyoto rats; SHR: spontaneous hypertensive rats; n/iCMIT: normal/increased carotid intima-media thickness.
